# Red Blood Cell Docosapentaenoic Acid (DPA *n*-3) is Inversely Associated with Triglycerides and C-reactive Protein (CRP) in Healthy Adults and Dose-Dependently Increases Following *n*-3 Fatty Acid Supplementation

**DOI:** 10.3390/nu7085291

**Published:** 2015-08-04

**Authors:** Ann C. Skulas-Ray, Michael R. Flock, Chesney K. Richter, William S. Harris, Sheila G. West, Penny M. Kris-Etherton

**Affiliations:** 1 Department of Nutritional Sciences, The Pennsylvania State University, University Park, PA 16802, USA; E-Mails: mike.flock@gmail.com (M.R.F.); ckr129@psu.edu (C.K.R.); sgw2@psu.edu (S.G.W.); pmk3@psu.edu (P.M.K.-E.); 2 OmegaQuant Analytics, LLC, Sioux Falls, SD 57106, USA; E-Mail: Bill@omegaquant.com; 3 Department of Biobehavioral Health, The Pennsylvania State University, University Park, PA 16802, USA

**Keywords:** fish oil, marine omega-3 fatty acids, DPA, inflammation

## Abstract

The role of the long-chain omega-3 (*n*-3) fatty acids eicosapentaenoic acid (EPA) and docosahexaenoic acid (DHA) in lipid metabolism and inflammation has been extensively studied; however, little is known about the relationship between docosapentaenoic acid (DPA, 22:5 *n*-3) and inflammation and triglycerides (TG). We evaluated whether *n*-3 DPA content of red blood cells (RBC) was associated with markers of inflammation (interleukin-6 (IL-6), tumor necrosis factor α (TNF-α), and C-reactive protein (CRP) and fasting TG prior to *n*-3 supplementation in two studies (Study 1: *n* = 115, aged 20–44 years, body mass index (BMI) 20–30 kg/m^2^, TG = 34–176 mg/dL; Study 2: *n* = 28, aged 22–65 years, BMI 24–37 kg/m^2^, TG = 141–339 mg/dL). We also characterized the dose-response effects of *n*-3 fatty acid supplementation on RBC *n*-3 DPA after five months of supplementation with fish oil (Study 1: 0, 300, 600, 900, and 1800 mg/day EPA + DHA) and eight weeks of prescription *n*-3 ethyl esters (Study 2: 0, 850, and 3400 mg/day EPA + DHA). In Study 1, RBC *n*-3 DPA was inversely correlated with CRP (*R*^2^ = 36%, *p* < 0.001) and with fasting TG (*r* = −0.30, *p* = 0.001). The latter finding was replicated in Study 2 (*r* = −0.33, *p* = 0.04). In both studies, *n*-3 supplementation significantly increased RBC *n*-3 DPA dose-dependently. Relative increases were greater for Study 1, with increases of 29%–61% *vs.* 14%–26% for Study 2. The associations between RBC *n*-3 DPA, CRP, and fasting TG may have important implications for the prevention of atherosclerosis and chronic inflammatory diseases and warrant further study.

## 1. Introduction

Long-chain omega-3 (*n*-3) polyunsaturated fatty acids (PUFA) reduce blood triglycerides [1,2] and play a role in preventing inflammatory diseases [3]. The relationships between intake and/or biomarkers of eicosapentaenoic acid (EPA, 20:5 *n*-3) and docosahexaenoic acid (DHA, 22:6 *n*-3) and blood concentrations of triglycerides and inflammatory markers have been studied extensively [4,5]. However, limited research has examined associations between these risk factors and docosapentaenoic acid (DPA, 22:5 *n*-3), which is also present in *n*-3 supplements in varying concentrations and is commonly consumed in seafood and red meat from ruminant animals [6]. 

Studies of plasma and serum *n*-3 DPA concentrations indicate that *n*-3 DPA also likely plays a role in influencing health outcomes that are responsive to EPA and DHA. Lower serum concentrations of DHA + *n*-3 DPA and *n*-3 DPA alone have been associated with greater risk of acute coronary events [7] and myocardial infarction [8], respectively. Plasma *n*-3 DPA was also inversely associated with total mortality (particularly from stroke-related deaths) [9], nonfatal myocardial infarction [10], and incident cardiovascular disease (CVD) in some ethnic groups [11]. Inverse associations have also been found between serum or plasma *n*-3 DPA and the C-reactive protein (CRP) [12,13]. 

Erythrocyte or red blood cell (RBC) fatty acid content may serve as a better biomarker than plasma for examining the relationship between *n*-3 DPA and disease risk factors. The fatty acid content of RBC membranes better reflects longer term dietary intakes than plasma and serum levels [14]. RBC membrane fatty acids also correlate with concentrations in other tissues/cells, and are associated with clinically relevant outcomes [15,16]. RBC fatty acids have been utilized extensively as a biomarker in studies of EPA and DHA [17], but few studies have reported relationships between RBC *n*-3 DPA and triglycerides or inflammatory markers despite the fact that *n*-3 DPA is present in RBC membranes in amounts comparable to EPA and DHA.

Little is known about how RBC *n*-3 DPA responds to *n*-3 supplementation. Limited evidence suggests that fish oil supplementation increases RBC *n*-3 DPA content [18]. Fish oil contains a relatively low concentration of DPA, and prior studies have not typically reported the DPA content of their supplements. Providing a complete characterization of the *n*-3 fatty acid profile of supplements would aid in the interpretation of RBC *n*-3 DPA responses. In addition to the *n*-3 DPA provided by supplements, endogenous production from other supplemented *n*-3 fatty acids, particularly EPA, may also be an important means of increasing tissue *n*-3 DPA reserves. For instance, plasma *n*-3 DPA increased significantly following supplementation with concentrated EPA ethyl esters [19,20] whereas no change in RBC *n*-3 DPA was found after three months of supplementation with a DHA-rich tuna oil [21]. A better understanding of how RBC *n*-3 DPA content responds to increased *n*-3 intake would provide insight needed to better characterize the role of *n*-3 DPA in the health effects attributed to *n*-3 fatty acids.

If RBC *n*-3 DPA levels are associated with triglycerides and inflammatory markers, and are responsive to *n*-3 supplementation, it is feasible that *n*-3 DPA may, in part, be responsible for some of the biological effects attributed to EPA and DHA. Thus, these evaluations are needed to increase our understanding of the potentially important biological effects of *n*-3 DPA. In order to evaluate the relationship between RBC *n*-3 DPA concentrations, triglycerides, and inflammation, we utilized data from two *n*-3 supplementation studies that enrolled healthy young adults (*n* = 115) and individuals with elevated triglycerides (*n* = 28) to examine associations between pre-supplementation RBC *n*-3 DPA content and markers of inflammation (interleukin-6 (IL-6), tumor necrosis factor α (TNF-α), and CRP and fasting triglycerides. We also evaluated the dose response effects of RBC *n*-3 DPA to *n*-3 supplementation in the two studies, which included different doses of fish oil (300, 600, 900, and 1800 mg/day EPA + DHA) administered for approximately five months and a prescription *n*-3 fatty acid ethyl ester concentrate (Lovaza™, 850 mg/day and 3400 mg/day EPA + DHA) administered for eight weeks to inform understanding of how increased *n*-3 intake affects endogenous stores of *n*-3 DPA.

## 2. Experimental Section 

### 2.1. Experimental Overview 

**Figure 1:**
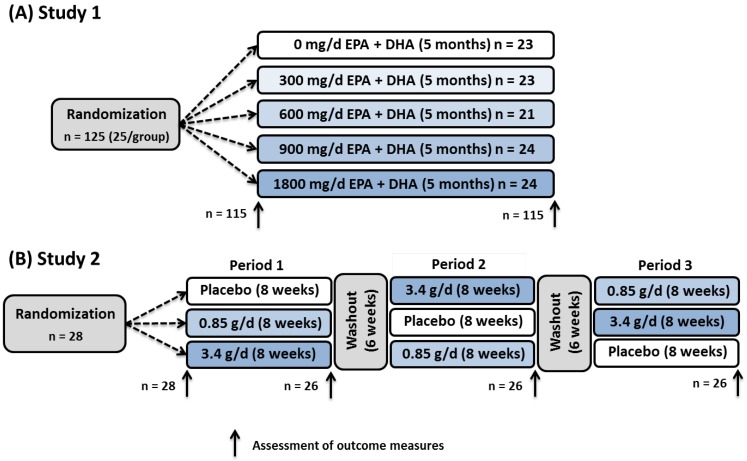
Study design schematic for Study 1 (**A**) and Study 2 (**B**). DHA, docosahexaenoic acid; EPA, eicosapentaenoic acid.

Experimental methods are described in detail in the original study reports [22,23]. Both studies (Figure 1) were registered on the web ClinicalTrials.gov (NCT01078909 and NCT00504309), conducted at the Clinical Research Center at the Pennsylvania State University, and approved annually by the Pennsylvania State University Institutional Review Board. 

In Study 1 [22] (Figure 1A; Table 1), healthy young adults (20–45 years of age, body mass index (BMI) 20–30 kg/m^2^, triglycerides (TG) 34–176 mg/dL) reporting no or low habitual oily fish consumption (<4 meals per month) and not taking *n*-3 PUFA supplements were randomly assigned to one of five doses of daily fish oil supplements (0, 300, 600, 900, 1800 mg EPA + DHA; soybean oil placebo) for approximately five months (Nordic Naturals, Watsonville, CA, USA). Of the 125 participants enrolled, 116 completed the study; however, one participant was excluded from analyses due to a baseline omega-3 index >8% (indicating high *n*-3 intake).

**Table 1:** Study design and baseline characteristics of participants who completed Study 1 and Study 2 ^1^.

	Study 1	Study 2
**Study design**	Parallel	Crossover
**Duration of supplementation**	20 weeks	8 weeks
**Total sample size**	115	26
**Male, *n*** (**%**)	60 (52%)	23 (88%)
**Age** (**year**)	26.1 ± 6.6 (20–45)	44.3 ± 9.8 (22–65)
**BMI** (**kg/m^2^**)	24.4 ± 2.5 (20–30)	29.0 ± 3.6 (23.7–36.5)
**Triglycerides** (**mg/dL**)	89.7 ± 32.1 (34–176)	222.8 ± 56.3 (140.5–339)
**CRP** (**mg/L**)	1.8 ± (<0.2–28.8)	1.3 ± 0.8 (<0.2–2.8)
**RBC *n*-3 DPA content**	2.4% ± 0.5 (1.3%–3.6%)	2.7% ± 0.4 (2.0%–3.5%)

^1^ Values are means ± standard deviation (SD) with ranges in parentheses; BMI, body mass index; CRP, C-reactive protein; RBC, red blood cells.

In Study 2 [23] (Figure 1B; Table 1), individuals who had moderately elevated triglycerides (141–339 mg/dL) but were otherwise healthy (22–65 years of age, BMI 24–37 kg/m^2^) and reported low habitual oily fish consumption (<2 servings/week) were enrolled in a three-period randomized crossover study comparing the effects of 850 and 3400 mg/day EPA + DHA to corn oil placebo. Each supplementation period lasted eight weeks and was separated by a six-week washout period. Twenty-eight participants were randomized to treatment groups and used for baseline analyses. Two participants did not complete the study, thus supplementation responses were assessed in 26 participants.

The long-chain *n*-3 fatty acid content provided by each supplement dose in Study 1 and Study 2, as determined by independent analysis, is provided in Table 2 [22,23]. 

**Table 2:** Omega-3 fatty acid content (mg/day) of each supplement dose in Study 1 and Study 2 ^1^.

	Study 1					Study 2		
	Dose (mg/day)					Dose (mg/day)		
**Fatty acid**	**0**	**300**	**600**	**900**	**1800**	**0**	**850**	**3400**
**Eicosapentaenoic acid (EPA)**	9	191	374	556	1103	0	486	1944
**Docosapentaenoic acid (DPA *n*-3)**	1	20	40	59	118	0	35	141
**Docosahexaenoic acid (DHA)**	6	121	237	352	698	0	421	1686
**Total EPA + DHA**	15	312	611	908	1801	0	907	3630
**Total EPA + DPA + DHA**	16	332	651	967	1919	0	942	3771

^1^ Values were calculated from independent analysis of fatty acid composition of a sample of active and placebo capsules.

### 2.2. Blood Sample Collection and Analysis

Both studies utilized the same methods for blood sample collection, measurement of inflammatory markers, and analysis of RBC fatty acid (FA) composition. At baseline and following treatment periods, a venipuncture blood sample was obtained from participants following a 12-h overnight fast. Whole blood was centrifuged at 1500× *g* for 15 min at 4 °C. Triglycerides were measured by enzymatic procedures (Quest Diagnostics, Pittsburgh, PA, USA; Coefficient of variation (CV) < 2%). Aliquots of serum and plasma were collected and stored at −80 °C until analysis. Serum concentrations of TNF-α and IL-6 were measured using high-sensitivity enzyme-linked immunosorbent assay (ELISA) kits (R&D Systems, Minneapolis, MN, USA) in duplicate (CV < 10%). Serum high-sensitivity CRP was measured by latex-enhanced immunonephelometry (Quest Diagnostics; assay CV < 8%). RBC were collected following separation from plasma by centrifugation and frozen at −80 °C prior to analysis. As previously described, RBC FA composition was analyzed by gas chromatography with flame ionization detection [24]. Briefly, lipids were extracted, methylated to form fatty acid methyl esters (FAMEs), and analyzed by gas chromatography on a GC2010 (Shimadzu Corporation, Columbia, MD, USA) equipped with a 100-m SP-2560 column (Supelco, Bellefonte, PA, USA). Fatty acids were identified by comparison with a standard mixture of FAs characteristic of RBCs (GLC 727; Nu-Check Prep, Waterville, MN, USA) that differentiated between *n*-3 and *n*-6 DPA isomers (Supplemental Figure S1). FAME composition was expressed as a percentage of total identified FAMEs (CV < 3.7%).

### 2.3. Statistical Analysis

Regression analyses for pre-supplementation associations were performed using Minitab (version 17, Minitab, State College, PA, USA). The assistant function was utilized to select between linear, quadratic, or cubic models. Residual *vs.* fit plots were examined to ensure homoscedasticity. To avoid extreme outliers due to acute infection in Study 1, baseline CRP values exceeding 10 mg/L (*n* = 3) were excluded from analysis. For Study 1, analysis of variance (ANOVA) was used to assess between group differences in RBC *n*-3 DPA responses. For Study 2, which was a crossover design, RBC *n*-3 DPA responses to supplementation were evaluated using the mixed models procedure (PROC MIXED) in SAS (Statistical Analysis System; version 9.3; SAS Institute Inc., Cary, NC, USA). Subject was treated as a random effect, and the effects of treatment, period, and treatment by period interactions were assessed. Period and treatment by period interactions were non-significant and removed from the final model of treatment effects. Tukey-adjusted *p*-values were used for *post hoc* comparisons between treatment groups.

## 3. Results 

### 3.1. Pre-Supplementation RBC n-3 DPA: Associations with Fasting Triglycerides and Markers of Inflammation, and Sex Differences

**Figure 2:**
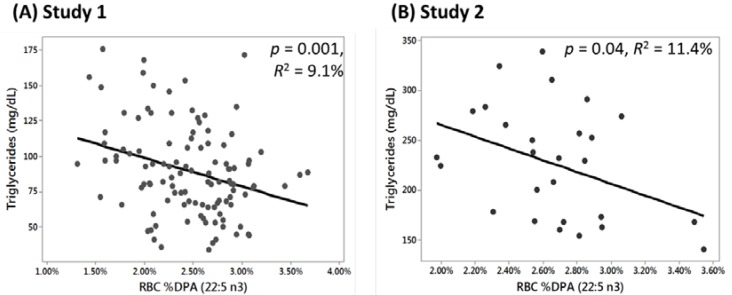
Scatterplots for regression analyses of serum triglycerides *vs.* red blood cell (RBC) % docosapentaenoic acid (DPA *n*-3) content at baseline (prior to supplementation). (**A**) RBC *n*-3 DPA values in Study 1 explained 9.1% of the variability in triglyceride values (*y* = 139.3 − 2022*x*); (**B**) In Study 2, RBC *n*-3 DPA values explained 11.4% of the variability in triglyceride values (*y* = 384 − 5922*x*).

Baseline RBC *n*-3 DPA content and fasting triglycerides were inversely associated in both Study 1 (Figure 2A, *r* = −0.30, *p* = 0.001) and Study 2 (Figure 2B, *r* = −0.33, *p* = 0.04). For every 1% increase in baseline RBC *n*-3 DPA, triglycerides were decreased by 20 mg/dL in Study 1 and by 59 mg/dL in Study 2. In both studies, baseline fasting triglycerides were not significantly related to the RBC content of EPA, DHA, or the Omega-3 Index (EPA + DHA, data not shown). 

**Figure 3:**
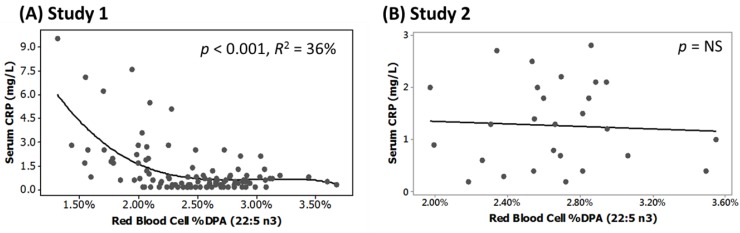
Scatterplots for regression analyses of serum C-reactive protein (CRP) *vs.* red blood cell (RBC) % docosapentaenoic acid (DPA *n*-3) content at baseline (prior to supplementation). (**A**) RBC *n*-3 DPA values in Study 1 explained 36% of the variability in serum CRP using a cubic fit (*y* = 32.43 − 3236*x* + 109213*x*^2^ − 12221024*x*^3^); (**B**) In Study 2, RBC *n*-3 DPA values were not significantly related to serum CRP.

In Study 1, baseline RBC *n*-3 DPA content and serum CRP concentrations were also inversely related with a cubic fit (*p* < 0.001) (Figure 3A). This model fit suggests a threshold for effects, such that RBC *n*-3 DPA is inversely related to CRP at *n*-3 DPA values <2% and/or higher CRP values. For *n*-3 DPA values >2%, there does not appear to be any predictive value of RBC *n*-3 DPA content with respect to CRP. There was no significant relationship between baseline RBC *n*-3 DPA and serum CRP in Study 2 (Figure 3B). No significant associations were found between RBC *n*-3 DPA content and the inflammatory cytokines IL-6 and TNF-α in Study 1 or Study 2 (data not shown). 

**Figure 4:**
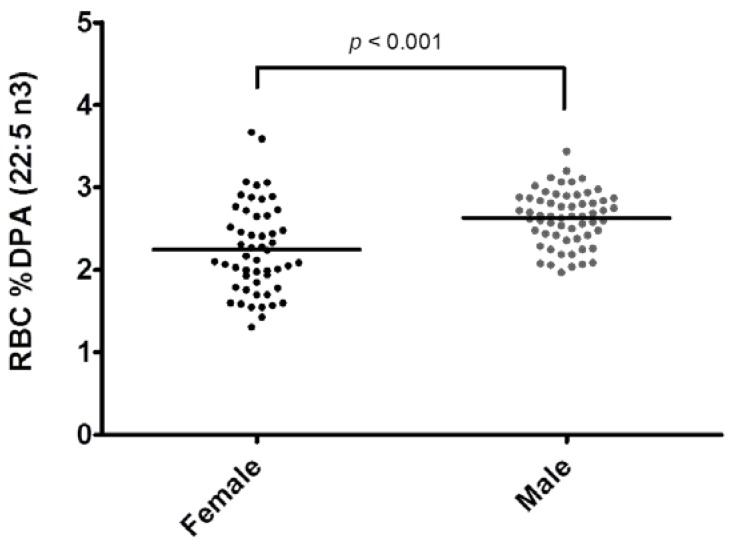
Sex differences in baseline red blood cell (RBC) % docosapentaenoic acid (DPA *n*-3) content.

Sex differences in baseline (pre-supplementation) RBC *n*-3 DPA content were also found, with male participants having approximately 0.38% higher RBC *n*-3 DPA content compared to female participants (Figure 4). 

**Figure 5:**
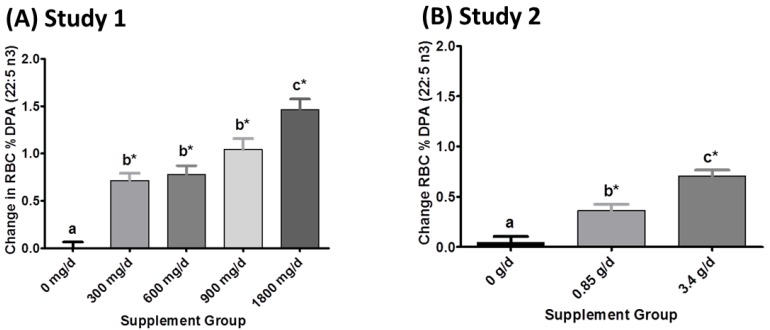
Mean (+/− standard error of the mean (SEM)) changes in red blood cell (RBC) docosapentaenoic acid (DPA *n*-3) content as a percentage of total fatty acids. Change scores for each treatment were calculated as end of treatment period value minus baseline value. Supplement groups refer to daily dose of Eicosapentaenoic acid (EPA) + docosahexaenoic acid (DHA) provided by one-gram oil capsules. (**A**) In Study 1, six capsules were consumed daily for five months; (**B**) In Study 2, four capsules were consumed daily for eight weeks. * Denotes significant difference *vs.* baseline. Different lowercase letters indicate significant differences between treatment groups, *p* < 0.05 (Tukey-adjusted *post hoc* pairwise comparisons).

### 3.2. Effects of Supplementation on RBC n-3 DPA Content

Five months of supplementation with 1 g to 6 g fish oil capsules providing 300–1800 mg EPA + DHA (Study 1) dose-dependently increased the percent of *n*-3 DPA in RBCs (Figure 5A). A significant 29% increase was observed for the lowest dose (1 g standard fish body oil containing 300 mg EPA + DHA and 20 mg *n*-3 DPA). The intermediate doses (600 mg/day and 900 mg/day EPA + DHA) similarly increased RBC *n*-3 DPA content. Following the high dose (6 capsules/day, providing 1800 mg/day EPA + DHA and 120 mg *n*-3 DPA), RBC *n*-3 DPA content increased 61% relative to the placebo group. RBC *n*-3 DPA content also increased dose-dependently in Study 2, but to a lesser extent. A 14% and 26% increase in RBC *n*-3 DPA occurred when 850 mg/day and 3400 mg/day EPA + DHA were administered as prescription *n*-3 ethyl ester capsules provided for eight weeks (Figure 5B).

## 4. Discussion 

To improve our understanding of the role of DPA in the health benefits derived from *n*-3 fatty acids, we performed secondary analyses of two published studies to examine the relationship between RBC *n*-3 DPA content, triglycerides, and inflammation, as well as the dose response effects of *n*-3 supplementation on RBC *n*-3 DPA stores. In healthy, younger adults, lower pre-supplementation RBC *n*-3 DPA concentrations were associated with higher levels of the inflammatory marker CRP. Lower RBC *n*-3 DPA was also associated with higher fasting triglycerides in these healthy adults and in individuals with moderately elevated triglycerides. Adjusting models for BMI, age, EPA, and DHA did not diminish the significance of RBC *n*-3 DPA as a predictor of CRP and triglycerides (data not shown). RBC EPA, DHA, or EPA + DHA did not significantly predict CRP or fasting triglycerides in these studies. RBC *n*-3 DPA exhibited a dose-dependent increase following *n*-3 supplementation that was proportionally greater following five months of supplementation compared to eight weeks of supplementation.

The inverse relationship between RBC *n*-3 DPA and triglycerides that we observed has previously been documented in only one population [25], and may have implications for the management of atherogenic dyslipidemia. Given the well-documented triglyceride-lowering effect of EPA and DHA supplementation, it is surprising that only RBC *n*-3 DPA was significantly related to fasting triglycerides in these two study populations and no relationship was found for EPA or DHA RBC content (both individually and in sum). We speculate that the lack of association may be related to the low habitual *n*-3 consumption of these participants, the conversion of dietary EPA to RBC *n*-3 DPA, and/or the influence of other factors that contribute to the development of high triglycerides. The efficacy of pure DPA supplementation for triglyceride reduction relative to EPA and DHA has not been studied. However, seal oil, a relatively rich source of DPA, has been shown to significantly reduce triglycerides in hypertriglyceridemic adults [26], with mixed results in healthy populations [27,28,29,30].

The inverse association between baseline RBC *n*-3 DPA content and serum CRP concentrations found in Study 1 agrees with prior studies [11,12,13,31,32] and may indicate a differential relationship between individual *n*-3 fatty acids and inflammatory markers. This inverse relationship with CRP has consistently been reported for plasma, serum, and RBC *n*-3 DPA concentrations [11,12,13,31,32]. In contrast, no association between CRP and RBC EPA or DHA content was found in the current study populations [4,23] or that of Labonte *et al.* [32]. Additionally, no relationship between RBC *n*-3 DPA content and inflammatory cytokines was found in either Study 1 or Study 2, despite there being an inverse relationship between RBC DHA and TNF-α in Study 1 [4]. EPA and DHA may promote resolution of inflammation via conversion into bioactive metabolites—resolvins, protectins, and maresins—collectively termed specialized pro-resolving mediators (SPMs). Novel *n*-3 DPA SPMs have also been characterized and demonstrated to have similar protective actions [33,34]. The anti-inflammatory effects of EPA may also in part be exerted via elongation to *n*-3 DPA as *n*-3 DPA produced from EPA supplementation was identified as the compound responsible for cyclooxygenase inhibition *in vitro* [35]. Based on the structural differences of *n*-3 DPA, EPA, DHA, and their metabolites, it is possible that they have unique effects in regulating different aspects of the innate immune system. The fatty acid composition of RBCs may also be differentially affected by the development of inflammation, and evaluating how RBC fatty acids are modified in the context of acute inflammation would serve to clarify this question. 

The lack of association between RBC *n*-3 DPA and CRP in Study 2 may be attributed to several factors. In Study 2, the range of CRP values was much narrower (all baseline values were <3 mg/L). This study also had a much smaller sample size that consisted predominately of middle-aged Caucasian males and included only three post-menopausal women (pre-menopausal women were excluded due to the difficulty of scheduling endpoint assessments at the same phase in each menstrual cycle). In Study 1, females had higher CRP values at baseline [4] and had significantly less RBC *n*-3 DPA content compared to male participants (Figure 4). These factors may have largely driven the observed relationship between CRP and RBC *n*-3 DPA. Women of all age groups currently consume very little DPA (as well as EPA and DHA) [36,37], and prior studies have similarly found lower serum [38], plasma [39], and RBC *n*-3 DPA [40] concentrations in women. Future studies should further examine sex differences in DPA stores and whether these relate to levels of inflammation, as women are at higher risk of many inflammatory diseases. 

Since *n*-3 DPA has been associated with inflammation [11,12,13,31,32] and several forms of cardiovascular disease [7,10,11,31], it is important to understand how *n*-3 DPA stores respond to *n*-3 supplementation. We found that RBC *n*-3 DPA content increased dose-dependently in both study populations. Significant increases were achieved even with the lowest dose (300 mg EPA + DHA), despite it providing only 20 mg/day *n*-3 DPA. The greatest increase was achieved following 1800 mg/day EPA + DHA (118 mg/day *n*-3 DPA) for five months. Although RBC *n*-3 DPA content increased significantly in both studies, the relative magnitude of change was much smaller in Study 2, despite larger doses of *n*-3 being provided (*i.e.*, 850 and 3400 mg/day compared to 300, 600, 900, and 1800 mg/day). This variance in RBC *n*-3 DPA responses may be due to differences in *n*-3 supplement preparation (*i.e.*, triglyceride *vs.* ethyl ester forms), differences in participant characteristics, and/or the duration of supplementation. Earlier work has not demonstrated a significant difference in bioavailability between these preparations, and we did not observe the same discordance in RBC EPA responses between Study 1 and Study 2. Given that the average RBC lifespan is ~120 days, it is likely that the longer supplementation period of Study 1 (*i.e.*, 20 weeks) allowed for greater RBC turnover, whereas in Study 2, only a portion of the original RBCs were replaced with *n*-3 supplemented RBCs during the eight-week supplementation period. RBC *n*-3 DPA—like DHA [41]—may also be relatively more stable over time and require a longer period of supplementation or dietary change to achieve maximal levels compared to EPA [42]. 

As the amount of *n*-3 DPA contained in these supplements was small, it is likely that endogenous conversion of EPA to DPA via elongation by fatty acid elongase-2 (FAE-2) and fatty acid elongase-5 (FAE-5) occurred [43]. Prior clinical studies have also found increases in RBC and plasma *n*-3 DPA following supplementation with EPA + DHA [18,26,42] and purified EPA [19,20,44]. The baseline association between EPA and *n*-3 DPA—but not *n*-3 DPA and DHA—in our study populations and others similarly supports this [9,10,31]. This relationship between the long-chain *n*-3 fatty acids is also supported by cell-based studies in which inter-conversion of EPA and *n*-3 DPA occurs readily [36,43,45], with limited conversion of *n*-3 DPA to DHA [46] and little evidence that DPA and DHA supplementation influence the production of one another [19,47,48]. Many have suggested that endogenous *n*-3 DPA stores are largely derived from the conversion of EPA based on poor correlations between plasma, serum, or RBC *n*-3 DPA and intake of fish or long-chain *n*-3 fatty acids of marine origin [9,10,11,31,32]. However, fish/seafood contains less *n*-3 DPA compared to EPA and DHA and more DPA is provided by ruminant meat sources [6,26,27]. Nutrient databases may also not currently provide adequate information about the DPA content of foods, similar to the lack of quantification of DPA content for most *n*-3 supplements. Although it has historically been difficult to obtain, pure DPA can be manufactured, and evaluating RBC fatty acid responses to pure EPA *vs.* pure DPA supplementation would help to clarify this relationship.

Furthermore, differences in the metabolism of the individual *n*-3 fatty acids and the inter-convertibility of *n*-3 DPA and EPA suggest that endogenous *n*-3 DPA reserves could serve as stores of EPA. In both cell-based [45] and clinical studies [47], DPA supplementation has been shown to increase EPA. There also appears to be cell/tissue specificity for *n*-3 storage as DHA is enriched in myocardial and neuronal membranes [49] and the EPA content of RBCs is lower than that of *n*-3 DPA and DHA [18,25,42,47]. It has been suggested that plasma EPA serves as a more dynamic and readily available pool of long-chain *n*-3 fatty acids that increases and decreases more quickly than DHA [10,41,42]. Thus, 22-carbon fatty acids may be preferentially stored in specific tissue compartments, and in the case of *n*-3 DPA, may serve to replenish plasma EPA that has been utilized. This may have important implications for the prevention or treatment of disease states known to be related to *n*-3 fatty acid intake and warrants further study. 

Detailed descriptions of the respective strengths and limitations of Study 1 and Study 2 have previously been discussed [4,22,23]. For the context of the current analysis, both studies used well-characterized *n*-3 supplements, and compliance rates were very high. Participants for both studies were also recruited according to strict inclusion/exclusion criteria and were required to be low fish consumers. However, no other dietary intake data was collected. As red meat is a major dietary source of DPA, this could have influenced baseline RBC *n*-3 DPA concentrations, but it is unlikely that participants drastically changed their meat consumption over the course of the study. Both studies included men and women, but Study 2 included only three post-menopausal women. The sample size of both studies was also relatively small, and the majority of participants were generally healthy Caucasians, which may limit the generalizability of these results. These analyses were strengthened by the wide distribution of baseline fasting triglyceride concentrations in both Study 1 and Study 2. However, the limited range of CRP values in Study 2 may have limited our ability to observe a significant relationship with RBC *n*-3 DPA content in this population. The design of both studies allowed for the opportunity to evaluate dose-response effects and provided insight into potential duration-response effects of *n*-3 supplementation on RBC *n*-3 DPA content. Both studies also utilized RBC fatty acids, a recognized biomarker of long-term dietary *n*-3 intake. 

## 5. Conclusions 

We have demonstrated that in both healthy adults and adults with moderately elevated triglycerides, RBC *n*-3 DPA content is associated with fasting triglycerides at baseline and dose-dependently increases in response to *n*-3 supplementation. Baseline CRP concentrations and RBC *n*-3 DPA were also inversely correlated in the population of healthy adults. Although this does not indicate causality, these results support the concept that *n*-3 DPA may play a role in the biological effects previously attributed solely to EPA and DHA. Further study is needed to replicate these findings in other populations and better characterize these relationships and the responsiveness of RBC *n*-3 DPA content to *n*-3 supplementation.

## Supplementary Files

Supplementary File 1
