# Supplementary material for: Red Blood Cell Docosapentaenoic Acid (DPA n-3) is Inversely Associated with Triglycerides and C-reactive Protein (CRP) in Healthy Adults and Dose-Dependently Increases Following n-3 Fatty Acid Supplementation
Source: Nutrients. 2015 Aug 4;7(8):6390–404. doi: 10.3390/nu7085291 (PMC4555130; doi:10.3390/nu7085291)
Supplement: Supplementary File 1 [file nutrients-07-05291-s001.docx]

**Supplementary Materials**


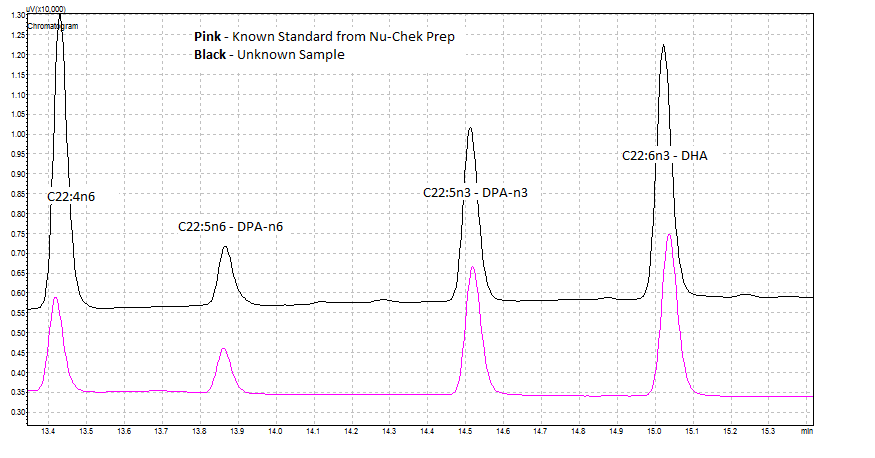


**Figure S1.** Differentiation of *n*-6 and *n*-3 docosapentaenoic (DPA) isomers in red blood cell (RBC) fatty acid analysis using a 100 m GC column.

© 2015 by the authors; licensee MDPI, Basel, Switzerland. This article is an open access article distributed under the terms and conditions of the Creative Commons Attribution license (http://creativecommons.org/licenses/by/4.0/).
